# Diabetes mellitus: knowledge and attitudes, collaborating for individual and social development of a reef community

**DOI:** 10.1186/1758-5996-7-S1-A174

**Published:** 2015-11-11

**Authors:** Tereza Cristina Pinho Paes Barreto, Amanda Pinho Paes Barreto, Joselí Reis, Rozângela Amorim Santos

**Affiliations:** 1 Pronto Socorro Cardiológico de Pernambuco, Recife, Brazil

## Background

The treatment of diabetes requires patient compliance, based on positive measures, inserted in social development.

## Objective

To compare the level of knowledge and the psychological adjustment to diabetes mellitus users of the Family Health Unit.

## Method

A prospective, cross-sectional, quantitative study, with comparison groups, carried out between June-August 2012 included 207 users of the Family Health Unit Finch Low, divided into three groups according to Results of glycated hemoglobin, analyzed by standard American Diabetes Association. The sample was divided into three groups as users were diabetic treated in the unit (group A=53); newly diagnosed diabetics in the unit (group B=85) and non-diabetic patients (group C=69). The collection tools included demographic information, anthropometric and related to the disease, the questionnaire of knowledge about diabetes (DKN-A) and psychological and emotional attitudes towards disease (TA-19). As well as adherence to behaviors related to individual and social development. The variables were organized using SPSS version 17.0 software and analyzed with the Student t test, Kolmogorov-Smirnov and chi square, at 0.05 significance level.

## Results

We diagnosed 41.06% of users as diabetic or pre-diabetic screening. Regardless of the group to which you belong, there was little knowledge about the disease, and negative psychological and emotional adaptation, pointing down user engagement to treatment.

## Conclusions

changes are needed in health education focused on diabetes, enabling formation of social consciousness that will motivate positive behavioral changes to patients and to society in general.

